# Local Inflammation Precedes Diaphragm Wasting and Fibrotic Remodelling in a Mouse Model of Pancreatic Cancer

**DOI:** 10.1002/jcsm.13668

**Published:** 2025-01-15

**Authors:** Daria Neyroud, Andrew C. D'Lugos, Enrique J. Trevino, Chandler S. Callaway, Jacqueline Lamm, Orlando Laitano, Brittney Poole, Michael R. Deyhle, Justina Brantley, Lam Le, Andrew R. Judge, Sarah M. Judge

**Affiliations:** ^1^ Department of Physical Therapy University of Florida Health Cancer Center Gainesville Florida USA; ^2^ Myology Institute University of Florida Gainesville Florida USA; ^3^ Institute of Sports Sciences University of Lausanne Lausanne Switzerland; ^4^ Department of Applied Physiology and Kinesiology University of Florida Gainesville Florida USA; ^5^ Department of Physiology and Aging, College of Medicine University of Florida Gainesville Florida USA

**Keywords:** cancer cachexia, inflammatory response, muscle atrophy, muscle fibrosis, pancreatic cancer

## Abstract

**Background:**

Cancer cachexia represents a debilitating muscle wasting condition that is highly prevalent in gastrointestinal cancers, including pancreatic ductal adenocarcinoma (PDAC). Cachexia is estimated to contribute to ~30% of cancer‐related deaths, with deterioration of respiratory muscles suspected to be a key contributor to cachexia‐associated morbidity and mortality. In recent studies, we identified fibrotic remodelling of respiratory accessory muscles as a key feature of human PDAC cachexia.

**Methods:**

To gain insight into mechanisms driving respiratory muscle wasting and fibrotic remodelling in response to PDAC, we conducted temporal histological and transcriptomic analyses on diaphragm muscles harvested from mice‐bearing orthotopic murine pancreatic (KPC) tumours at time points reflective of precachexia (D8 and D10), mild–moderate cachexia (D12 and D14) and advanced cachexia (endpoint).

**Results:**

During the precachexia phase, diaphragms showed significant leukocyte infiltration (+3‐fold to +13‐fold; D8—endpoint vs. Sham, *p* < 0.05) and transcriptomic enrichment of inflammatory processes associated with tissue injury that remained increased through endpoint. Diaphragm inflammation was followed by increases in PDGFR‐ɑ^+^ fibroadipogenic progenitors (+2.5 to +3.8‐fold; D10—endpoint vs. Sham, *p* < 0.05), fibre atrophy (−16% to −24%, D12 to endpoint vs. Sham, *p* < 0.05), ECM expansion (+1.5 to +1.8‐fold; D14—endpoint vs. Sham, *p* < 0.05), collagen accumulation (+3.8‐fold; endpoint vs. Sham, *p* = 0.0013) and reductions in breathing frequency (−55%, *p* = 0.0074) and diaphragm excursion (−43%, *p* = 0.0006). These biological processes were supported by changes in the diaphragm transcriptome. Ingenuity pathway analysis predicted factors involved in inflammatory responses to tissue injury, including TGF‐β1, angiotensin and PDGF BB, as top upstream regulators activated in diaphragms prior to and throughout cachexia progression, while PGC‐1α and the insulin receptor were among the top upstream regulators predicted to be suppressed. The transcriptomic dataset further revealed progressive disturbances to networks involved in lipid, glucose and oxidative metabolism, activation of the unfolded protein response and neuromuscular junction remodelling associated with denervation.

**Conclusions:**

In summary, our data support leukocyte infiltration and expansion of PDGFRα mesenchymal progenitors as early events that precede wasting and fibrotic remodelling of the diaphragm in response to PDAC that may also underlie metabolic disturbances, weakness and respiratory complications.

## 1 Introduction

Cancer cachexia is a debilitating condition characterised by the involuntary loss of lean body mass, with or without concomitant loss of fat mass [1]. This cancer‐associated pathology contributes to reduced functional capacity and quality of life in patients and limits cancer treatment options and efficacy [2]. Cachexia is estimated to contribute to ~30% of cancer‐related deaths, with deterioration of cardiorespiratory muscles suspected to be a major contributor to cachexia‐associated morbidity and mortality [3]. While cachexia can be triggered by multiple types of cancer, it is highly prevalent in patients with pancreatic cancer [4], with up to 70% of patients presenting with body weight loss of 5% or more at the time of diagnosis [5].

In recent work, we demonstrated that patients with pancreatic ductal adenocarcinoma (PDAC) exhibiting cachexia showed significant morphological disruptions in skeletal muscle biopsied from the *rectus abdominis*—a respiratory accessory muscle involved in forceful expirations. In this regard, we observed evidence of muscle damage, inflammation, degeneration/regeneration and expansion of muscle connective tissue, including fat and fibrotic tissue [6]. Notably, increased muscle fibrotic tissue was associated with regional lymph node metastasis and was identified as an independent predictor of survival in PDAC patients, thereby linking respiratory muscle fibrosis to poor outcomes in this patient population.

Several studies have now shown an increased presence of immune cells in skeletal muscle tissues of cachectic tumour‐bearing mice [7, 8] and humans [6, 9, 10], with a handful of studies also demonstrating increased deposition of extracellular connective tissue [6, 11, 12]. Data from our lab using the preclinical PDAC patient‐derived xenograft (PDAC‐PDX) model further found that mouse respiratory muscles better replicate human PDAC cachexia pathophysiology than peripheral limb muscles [12], at least when comparing to human biopsies from the *rectus abdominis*. In this regard, similar to PDAC patients exhibiting cachexia, we identified that the diaphragm of cachectic PDAC‐PDX mice showed increased deposition of collagen and transcriptional changes supporting immune cell trafficking, inflammation and connective tissue expansion [12]. These similarities suggest that studying the response of the mouse diaphragm to PDAC may offer novel insights into the response of human respiratory muscles to PDAC as it relates to cachexia and pathological muscle remodelling. Herein, we therefore sought to better define the temporal changes impacting the mouse diaphragm in response to PDAC prior to, and throughout, cachexia development and progression.

## 2 Methods

### 2.1 Cells

Pancreatic KPC FC1245 tumour cells were a kind gift of Dr. David Tuveson (Cold Spring Harbour Laboratory, Cold Spring Harbour, New York, NY). These cells were derived from a LSL‐Kras^G12D/+^; LSL‐Trp53^R172H/+^; Pdx‐1‐Cre mouse backcrossed to the C57BL/6 genetic background and were previously described [13]. KPC FC1245 cells were cultured in Dulbecco's Modified Eagle Medium (Thermo Fisher Scientific, Waltham, Massachusetts, USA) supplemented with 10% foetal bovine serum (Thermo Fisher Scientific), 1% penicillin (Thermo Fisher Scientific) and 1% streptomycin (Thermo Fisher Scientific) at 37°C in a 5% CO_2_ humidified chamber.

### 2.2 Animals

C57Bl/6 J male mice were purchased from The Jackson Laboratory (Bar Harbor, Maine, USA). All animal procedures were approved by the University of Florida Institutional Animal Care and Use Committee (IACUC). Mice were provided with ad libitum access to food and water and were housed in a temperature‐ and humidity‐controlled facility on a 12 h dark/light cycle. The pancreas of ten‐week‐old male C57BL/6 J mice was surgically exposed, and 0.25 × 10^6^ cells diluted in 50 μL of sterile PBS (KPC group) or 50 μL of sterile PBS (Sham group) were orthotopically injected as previously described [14]. To evaluate the progression of KPC‐induced cachexia, tissues were harvested from KPC tumour‐bearing mice at various time points (*N* = 6 per time point), including on Day 8 (D8), Day 10 (D10), Day 12 (D12) and Day 14 postsurgery (D14), and at IACUC‐mandated endpoint (i.e., 15 to 18 days postsurgery, END) based on body condition score and/or body mass loss. Sham mice were euthanised on D8, D14 and D15.

### 2.3 In Vivo Diaphragm Ultrasound Imaging

In vivo diaphragm function was evaluated via M‐mode ultrasound imaging (V LOGIQ e Vet NEXTGEN, GE, Boston, MA, USA) immediately prior to euthanasia while mice were maintained under light anaesthesia (2% isoflurane in 98% O_2_) with an O_2_ flow set at 1.5 L/min as previously described [15]. After shaving and cleaning of the sternum/abdominal area, mice were placed in a supine position with a 2.5–7 phased array transducer positioned horizontally below the diaphragm immediately distal to the xiphoid process. At least two 10‐s M‐mode video sequences were recorded and used to measure diaphragm excursion amplitude (i.e., vertical displacement) and breathing frequency and to estimate minute ventilation (i.e., diaphragm excursion amplitude x breathing frequency). A total of six breaths were analysed and averaged per animal.

### 2.4 Blood Collection

Blood from the inferior *vena cava* artery was collected, mixed with clotting factors (Microtainer BD 365967, BD, Franklin Lakes, New Jersey, USA), incubated at room temperature for at least 30 min and centrifuged at 2500 g for 10 min at 4°C. Serum samples were then stored at −80°C until analysis.

### 2.5 Cytokine and Chemokine Analyses

Serum circulating factors were screened using a 25‐plex mouse cytokine/chemokine magnetic bead panel (25 Plex MILLIPLEX MAP Mouse Cytokine/Chemokine Magnetic Bead Panel, Millipore, Burlington, Massachusetts, USA) as previously described [16], following the manufacturer recommendations.

### 2.6 Immunohistochemistry

Upon harvest, tissues were embedded in optimal cutting temperature compound (OCT) and frozen in liquid isopentane cooled in liquid nitrogen before storing at −80°C. Samples were equilibrated at −20°C before cutting 10‐μm sections using microtome cryostat. Sections were dried and stored at −80°C. Prior to staining, sections were thawed and air‐dried for 30 min at room temperature. Haematoxylin and eosin (H&E) staining was performed as previously described (Supporting Information Reference 1). For fibre‐type‐specific myofiber size, sections were blocked for 60 min with 10% normal goat serum at room temperature and incubated for 90 min with antibodies against myosin heavy chain I (BA‐D5, DSHB; University of Iowa, Iowa, USA) and myosin heavy chain IIA (SC‐71, DSHB) at a concentration of 1:10 in PBS at room temperature and incubated for 1 h with appropriate fluorescently conjugated secondary antibodies (#A21141, #A21120, Invitrogen, Carlsbad, CA, USA) and Alexa Fluor 594‐conjugated wheat germ agglutinin (WGA) (Invitrogen) at room temperature. Total collagen content was determined through picrosirius red staining, where slides were fixed in Bouin's fluid and incubated for 90 min in picrosirius red solution (0.1% Direct Red 80 in saturated picric acid) prior to dehydration and cover slipping. The extent of collagen remodelling was assessed by staining cross‐sections with a collagen hybridising peptide (CHP; 3Helix, Salt Lake City, Utah, USA) as previously described [17]. Leukocyte infiltration and FAP expansion were quantified as described in [18] following immunofluorescent staining using antibodies against CD45 (a pan leukocyte marker; #553076, BD Bioscience, New Jersey, USA) and PDGFRɑ (i.e., a FAP marker; A32814, Invitrogen), respectively. All sections were imaged with a widefield Leica DM5000B or confocal Leica TCS SP8 microscope (Leica, Microsystems, Bannockburn, Illinois, USA). ImageJ was used for all analyses (Supporting Information Reference 2). Skeletal muscle fibre size was measured by a semi‐automated threshold analysis as previously described [19]. A total of 420–2170 (mean: ~ 1200) fibres were traced for each diaphragm cross‐section. The WGA signal was also used to detect changes in extracellular matrix (ECM)/fibrosis, by quantifying the percentage of the muscle area occupied by WGA‐positive staining (Supporting Information Reference 3). The degree of collagen remodelling was assessed by quantifying the percentage of muscle area occupied by CHP‐positive staining [17]. The number of CD45 positive cells was calculated by manually counting CD45^+^/DAPI^+^ cells. The degree of FAP expansion was determined by quantifying the percentage of the muscle area occupied by PDGFRɑ‐positive staining.

### 2.7 RNA Isolation

RNA isolation was performed as described previously [12]. Briefly, muscle tissues were homogenised in Trizol (Thermo Fisher Scientific), and RNA was extracted with phenol/chloroform protocol and treated with DNAse (AM1906, Invitrogen).

### 2.8 RNA Sequencing and Analysis

RNA sequencing analyses were performed by Novogene (Novogene Co, Ltd., Sacramento, California, USA). Briefly, RNA degradation and contamination were evaluated on 1% agarose gel, RNA purity was checked using a NanoPhotometer spectrophotometer (Implen Inc., Westlake, California, USA) and RNA integrity and quantity were assessed using an RNA Nano 6000 Assay and a Bioanalyzer 2100 system (Agilent Technologies, Santa Clara, California, USA). A total of 1 μg of RNA per sample and three biological replicates per group were used to generate sequencing libraries. Paired‐end clean reads were aligned to the reference genome using the Spliced Transcripts Alignment to a Reference (STAR) software (version 2.5). Fragment Per Kilobase of exon model per Million mapped reads (FPKM) were calculated using the HTSeq package (version 0.6.1) based on the length of the gene and reads count mapped to this gene. Follow‐up analyses were conducted using R (version 4.2.3) in the RStudio Integrated Development Environment (version 2023.03.0+386). Principal component analysis (PCA) was conducted using the R built‐in prcomp function on log2‐transformed, scaled and normalised read counts. Differential expression between groups was calculated using the DESeq2 R package, and the resulting *p*‐values were adjusted using the Benjamini and Hochberg's approach. A hierarchical clustering analysis was performed on differentially expressed genes (adjusted *p*‐value < 0.01) using the WardD2 method on variance stabilised data. Bioinformatic enrichment analyses were conducted using Ingenuity Pathway analysis (IPA, QIAGEN Inc., https://digitalinsights.qiagen.com/IPA; Supporting Information Reference 4), DAVID (version 2021, Supporting Information Reference 5, 6) and Enrichr (Supporting Information Reference 7–9). At each time point, upstream regulator analyses were performed using IPA, while DAVID was used to identify enriched biological processes and pathways. Bioinformatic enrichment analyses performed on genes within hierarchical clusters were conducted with DAVID to identify enriched biological processes and KEGG pathways and Enrichr to identify potential upstream transcriptional regulators and cell type signatures.

### 2.9 Statistical Analyses

Shapiro–Wilk tests were used to test data normality. Depending on Shapiro–Wilk test outcome, parametric or nonparametric tests were used. One‐way ANOVAs were performed to examine the effect of increased tumour burden on body mass and changes in body mass, tissue mass (except for skeletal muscle, see below), respiratory rate, diaphragm excursion, minute ventilation, mean fibre diameter, muscle fibre typology, percent area occupied by WGA‐positive staining, percent area occupied by CHP‐positive staining, CD45^+^ cell number and cytokine and chemokine concentrations. CD45^+^ cell number data were also fit with an exponential model. The effect of tumour burden on skeletal muscle mass was evaluated using a nested one‐way ANOVA. When a statistically significant difference was detected by the ANOVA, Dunnett (for one‐way ANOVA) or Dunn's (for Kruskal–Wallis one‐way ANOVA) post hoc was used to test for differences among pairs of means. The alpha level for statistical significance was set a priori to 0.05. All data are reported as mean ± standard error. Apart from the RNAseq, all statistical analyses were performed using Prism (version 10.0.0, GraphPad Software, La Jolla, California, USA).

## 3 Results

### 3.1 Cachexia Development and Progression in an Orthotopic KPC Model

**FIGURE 1:**
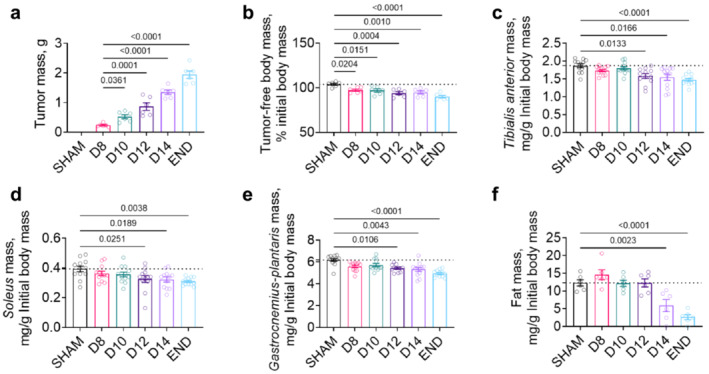
Orthotopic KPC tumours induce progressive body wasting associated with declines in skeletal muscle, heart and fat mass. Orthotopic injection of murine pancreatic cancer (KPC) cells induces exponential growth of KPC tumours (a) and causes progressive declines in body mass (b) associated with wasting of skeletal muscles (c–e) and fat (f). Cachexia, based on significant wasting of skeletal muscle tissues and reduced body mass, was first observed on D12. All *p*‐values < 0.1 are included, symbols represent individual data and bars represent mean ± SE.

To provide a comprehensive time course reflecting the different stages of PDAC cachexia, mice were orthotopically inoculated with murine pancreatic cancer (KPC) cells (Supporting Information Figure S1a). KPC tumour‐bearing mice showed exponential tumour growth (*r* ^2^ = 0.8709, Figure 1a and Supporting Information Figure S1b) and progressive tumour‐free body mass reduction (Figure 1b). Muscle wasting was significant from day 12 (D12) onwards (Figure 1c–e) and cancer cachexia—marked by a concomitant loss of body and skeletal muscle mass [1]—was thus first detected 12 days following KPC cell inoculation. Fat and cardiac wasting were observed by day 14 (D14; Figure 1f and Supporting Information Figure S1c). However, the sham surgery alone causes significant fat wasting (Supporting Information Figure S2), which could potentially mask early cancer‐related losses in fat mass. Lastly, KPC tumour burden induced splenomegaly (Figure S1d) which is indicative of systemic inflammation. Liver mass was not statistically changed in KPC mice at any time point (Figure S1e). Multiplex analyses performed on serum revealed a transient increase in the abundance of chemoattractant factor keratinocyte chemoattractant (KC) and interleukin‐5 (IL‐5) in precachectic mice (D8 and D10, Figure S3a,b). At the time of cachexia onset (D12), interleukin‐6 (IL‐6) showed increased abundance, while interferon gamma‐induced protein 10 (IP‐10) showed reduced abundance (Figure S3c‐d). IL‐6 remained numerically increased at D14 and statistically increased at endpoint (END, Figure S3c). Granulocyte colony stimulating factor (G‐CSF) levels were statistically elevated at endpoint only (Figure S3e). Overall, our data demonstrate that pancreatic tumours cause systemic inflammation that develops prior to cachexia onset and remains throughout cachexia progression.

### 3.2 KPC‐Induced Ventilatory Modifications

**FIGURE 2:**
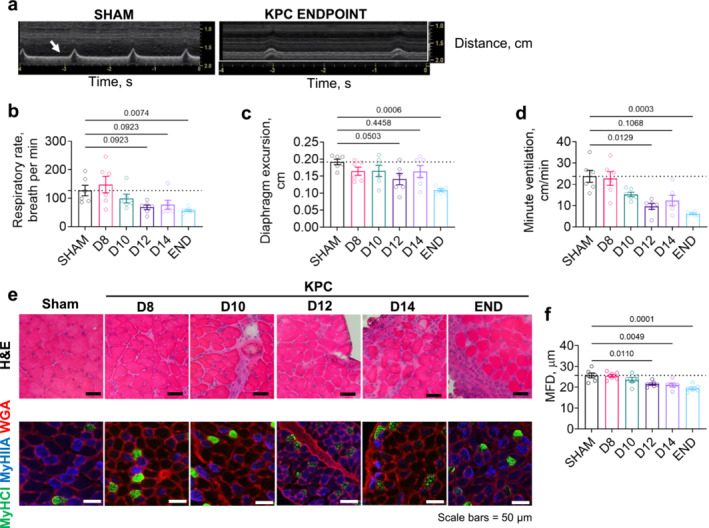
Reductions in breathing frequency and diaphragm excursion are associated with progressive diaphragm myofiber atrophy and alterations in fibre‐type composition. (a) Representative ultrasonographic traces depicting diaphragm movements recorded in a nontumour bearing Sham mouse and a mouse bearing a pancreatic (KPC) tumour at humane endpoint. (b‐d) KPC tumour burden leads to reductions in respiratory rate (b), diaphragm excursion (c) and estimated minute ventilation (d). (e) Representative images of diaphragm cross‐sections stained with haematoxylin and eosin (H&E) or with antibodies against wheat germ agglutinin (WGA) to label muscle fibre borders and myosin heavy chain I (MyHCI) and myosin heavy chain IIA (MyHCIIA) to distinguish between Type I (green), Type IIA (blue) and Type IIX/B (black) myofibers. (f) Quantification of diaphragm minimum Feret diameter (MFD) reveals KPC‐induced diaphragm fibre atrophy beginning at the time of cachexia onset (D12). All *p*‐values < 0.1 are included, symbols represent individual data and bars represent mean ± SE.

Respiratory complications are highly prevalent among patients with late‐stage cancer [20]—a stage at which the large majority of patients present with cachexia [21]. Although multiple factors may contribute to these complications, disruptions to diaphragm architecture and function could play a role. Recent work showed that tumour burden causes significant reductions in ex vivo diaphragm specific force in the C26 model prior to and throughout cachexia development [22], while work from our lab demonstrated significant diaphragm weakness in the orthotopic KPC model in moderately and severely cachectic mice [19, 23]. Herein, we used ultrasonography to noninvasively examine time‐dependent changes in diaphragm function, in vivo, through measurement of diaphragm excursion amplitude (Figure 2a). Compared to Sham mice, KPC mice showed progressive reductions in breathing frequency (Figure 2b) and diaphragm excursion amplitude (Figure 2c) throughout the cachexia trajectory, reaching statistical significance at endpoint. Note that diaphragm excursion amplitude has been shown to strongly correlate with ex vivo diaphragm specific force values and to negatively correlate with diaphragm fibrosis in a mouse model of muscular dystrophy [15]. As a result of the decline in breathing frequency and reduced diaphragm excursion, a progressive reduction in minute ventilation may be predicted as early as D10 (Figure 2d).

### 3.3 Leukocyte Infiltration and Expansion of PDGFRα Mesenchymal Progenitors Precedes Muscle Fibre Atrophy and ECM Remodelling in the Diaphragm of KPC Mice

We previously observed both muscle fibre atrophy and structural alterations within the diaphragm of preclinical PDAC models at endpoint [7, 12], which may contribute to diaphragm dysfunction. Analysis of muscle fibre minimum Feret diameter (MFD) indicates that diaphragm fibre atrophy is observed from the time of cachexia onset (D12) in KPC mice (Figure 2e–f). Fibre typology assessment further revealed a predominant atrophy of type IIA and type IIX/B fibres, with no significant changes in type I myofibers (Figure S4a–c). We also observed a reduction in the percentage of type IIX/B myofibers and a corresponding increase in the percentage of type I and IIA myofibers after cachexia onset (Figure S4d–f).

**FIGURE 3:**
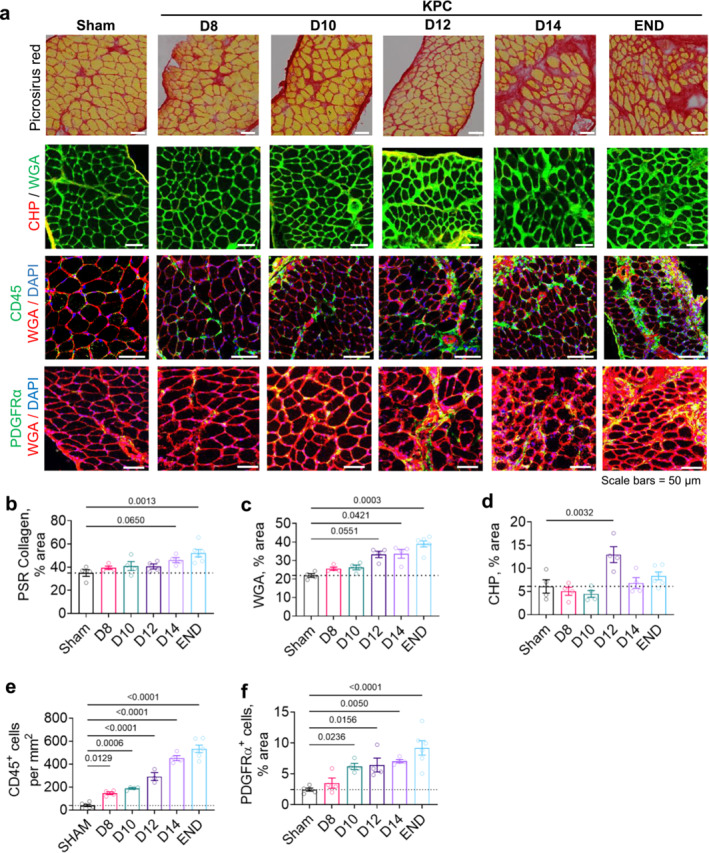
Immune cell infiltration and expansion of FAPs precede KPC‐induced diaphragm atrophy and fibrotic remodelling. (a) Representative images of diaphragm cross‐sections from Sham and KPC mice (D8, D10, D12, D14, END) stained with Picrosirius red to label collagen (red), a collagen hybridising peptide (CHP) to label unfolded/degraded collagen, an anti‐CD45 antibody to label leukocytes and an anti‐PDGFRɑ antibody to label fibroadipogenic progenitors (FAPs). Note that some cross‐sections were co‐stained with DAPI to label nuclei and wheat germ agglutinin (WGA), which labels glycosylated proteins abundant in myofiber membranes and in the extracellular matrix. (b) KPC tumours induce significant deposition of collagen at endpoint (END) that is preceded by extracellular matrix expansion (% WGA+ area) (c) and collagen damage/remodelling (% CHP+ area) (d). (e) Increased numbers of CD45+ leukocytes are observed in diaphragm muscles of KPC mice as early as D8, prior to cachexia development and extracellular matrix remodelling, and show an exponential (*r* ^2^ = 0.9126) increase thereafter. (f) Expansion of PDGFR+ FAPs in diaphragm muscles of KPC mice is observed during the precachectic stage on D10, following leukocyte infiltration, and progressively increases through endpoint. All *p*‐values < 0.1 are included, symbols represent individual data and bars represent mean ± SE.

H&E staining of diaphragm muscles from KPC mice revealed a progressive pathology similar to that observed in muscles of cachectic PDAC patients [6], including increased presence of mononuclear cells, myofibers with either diffusely dark or pale cytoplasmic staining (indicative of damage & necrosis) and increased presence of connective tissue (Figure 2e). Picrosirius red and wheat germ agglutinin (WGA, Figure 3a) staining further showed ECM expansion from D12 onwards, with total muscle area staining positive for collagen trending toward significance on D14 and significantly elevated at endpoint (Figure 3b,c). As collagen damage and degradation is an early and transient event that precedes the development of tissue fibrosis, we also stained diaphragm cross‐sections with a CHP, which uniquely binds to unfolded collagen in conditions of inflammation and tissue damage (Figure 3a) [24]. Binding of CHP within diaphragms of KPC mice transiently increased by twofold at the time of cachexia onset (i.e., D12, Figure 3d) but was not significantly changed at any later time points. This finding suggests that factors that degrade collagen, such as metalloproteinases (MMPs), may predominate during cachexia onset, while factors promoting collagen accumulation may dominate during the later stages of cachexia, when increased collagen is observed.

ECM expansion and collagen deposition in muscle is a normal transient response to tissue injury that stabilises the contractile apparatus during regenerative processes. However, a number of factors—including systemic inflammation related to underlying disease [25] and persistence of an injury stimulus—can result in chronic tissue inflammation and leukocyte presence that impairs tissue regeneration, leading to sustained ECM accumulation and fibrosis development [26]. In the context of cancer cachexia, leukocyte infiltration into muscle and muscle fibrosis have both been observed in response to multiple cancer types [6, 7, 8, 9, 10, 11, 12]. Furthermore, signalling through the inflammatory transcription factor, NF‐kB—which can be activated by pro‐inflammatory cytokines—has been established to inhibit satellite cell differentiation, impeding muscle regenerative capacity and mediating cancer‐induced muscle loss [27]. Together these results suggest that local tissue inflammation may be a key upstream trigger of muscle wasting and ECM remodelling in response to cancer. We therefore further stained diaphragm tissue cross‐sections with a CD45 antibody (a pan leukocyte marker) to label leukocytes (Figure 3a). We observed an early (D8) and exponential increase in CD45^+^ cells throughout the development and progression of cachexia (Figure 3e), supporting leukocyte infiltration as an early event that precedes cachexia. We subsequently stained diaphragm muscles for PDGFR‐α (Figure 3a), a marker of mesenchymal progenitors that labels skeletal muscle fibroadipogenic progenitors (FAPs) which expand following posttraumatic inflammation. Depending on niche signals, FAPs may differentiate into either adipocytes or fibroblasts, contributing to fat and/or fibrotic tissue accumulation within muscle [28]. Previous work has shown that in limb muscles of cachectic mice‐bearing C26 tumours, PDGFRα is also expressed by Pax7+ cells of nonsatellite cell origin that accumulate in cachectic muscle, yet fail to differentiate [27]. Similar to our previous observations in the *rectus abdominis* muscle of PDAC patients [6], we found that PDGFR‐α^+^ mesenchymal progenitors were significantly increased in diaphragm muscles of KPC mice by D10, and remained increased through endpoint (Figure 3f). Collectively, these data support leukocyte infiltration into the diaphragm of tumour‐bearing mice as an early event that precedes PDGFR‐α+ progenitor expansion, with both processes occurring prior to muscle atrophy and fibrotic remodelling.

### 3.4 Transcriptomic Analyses Support Immune Cell Trafficking and Cytokine Signalling as Early Events That Precede Diaphragm Wasting and Fibrotic Remodelling

To gain insight into the molecular mechanisms driving diaphragm wasting and pathological remodelling in response to pancreatic tumour burden, diaphragms harvested from Sham mice and KPC mice at each time point were subjected to RNA‐seq analyses. PCA revealed good separation between time points (Figure S5). As tumours grew and cachexia developed and progressed, we observed a progressive increase in the number of differentially expressed genes (DEG, padj. < 0.01, Supporting Information File S1). In total, 352 (D8), 860 (D10), 1353 (D12), 1795 (D14) and 3060 (END) showed a > twofold upregulation, while 58 (D8), 282 (D10), 1011 (D12), 1284 (D14) and 2759 (END) showed a > twofold downregulation at each time point, respectively.

**FIGURE 4:**
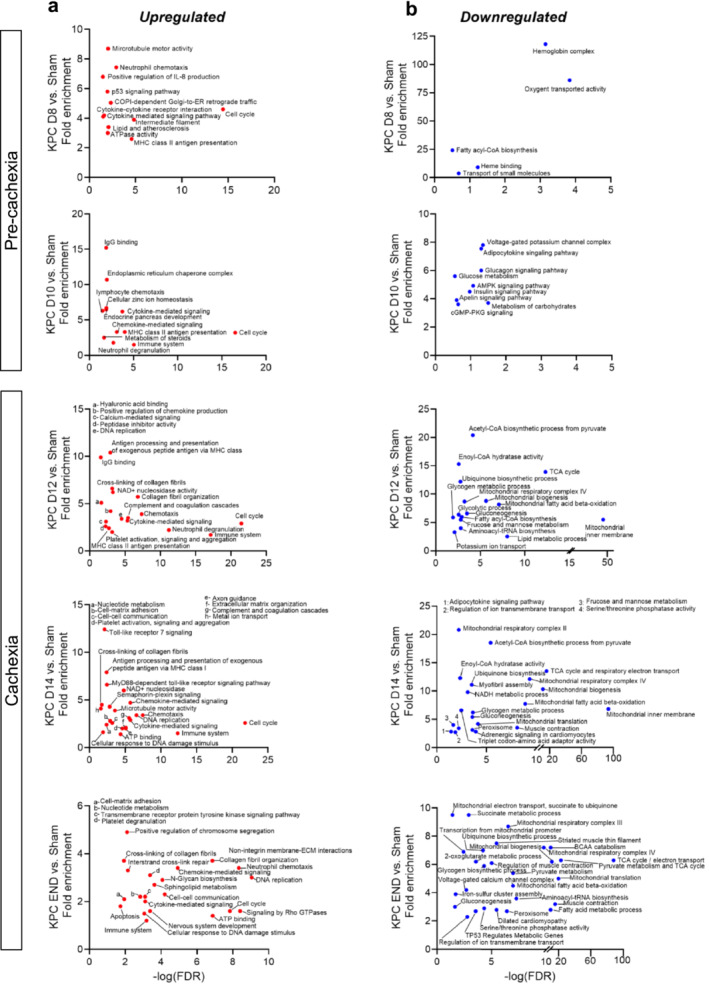
Biological processes and pathways enriched in the diaphragm throughout the development and progression of KPC‐induced cachexia. RNA sequencing was conducted on diaphragm from Sham mice and KPC mice at time points reflective of precachexia (D8, D10) and cachexia (D12, D14, END). Bioinformatic enrichment analyses using the DAVID platform were performed on genes demonstrating a significant (padj. < 0.01) twofold upregulation (a) or downregulation (b) in diaphragms of KPC mice (vs. Sham) at each time point.

**FIGURE 5:**
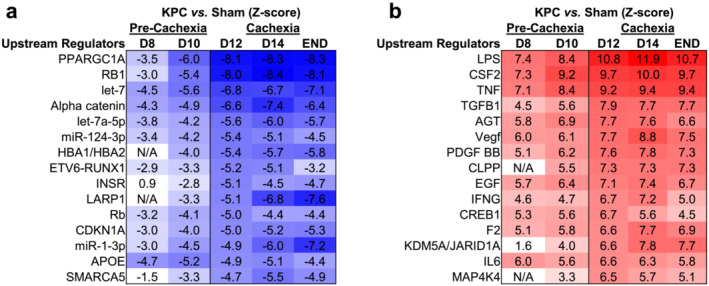
Ingenuity Pathway Analysis (IPA) of diaphragm RNAseq data identifies top upstream regulators predicted to drive transcriptional changes throughout the progression of PDAC cachexia. Top 15 upstream regulators predicted by IPA to be suppressed (a) or activated (b) in diaphragm muscles of KPC mice (vs. Sham) at the time point when cachexia and diaphragm atrophy are first observed (D12). *Z*‐scores are shown for upstream regulators at all time points, including those reflective of precachexia (D8 and D10) and cachexia (D12, D14 and END).

To investigate biological processes and pathways altered during the different phases of cachexia, genes significantly upregulated or downregulated (padj. < 0.01 and twofold change vs. Sham) at each time point were analysed via bioinformatic enrichment analyses (Figure 4 and Supporting Information File S2). At the earliest precachexia time point (D8) when leukocyte infiltration was observed, upregulated genes were enriched for processes related to cell cycling, immune cell trafficking and cytokine signalling, as well as lipid and atherosclerotic processes and included marker genes for monocytes (*Cd14*) and macrophages (*Cd68*) (Figure 4a). Inflammatory processes remained highly upregulated throughout the development and progression of cachexia (Figure 4a), with genes related to neutrophil degranulation enriched by D10 and platelet activation and aggregation by D12. On D10, the time point immediately preceding cachexia development, upregulated genes was enriched for not only inflammatory pathways but also for pathways related to sterol/cholesterol metabolism, endoplasmic reticulum (ER) stress and zinc ion homeostasis. At the time of cachexia onset (D12), when we first observed fibre atrophy and ECM expansion, upregulated genes showed enrichment of pathways related to ECM remodelling and fibrosis and included markers for anti‐inflammatory M2 macrophages (*Cd163*) which are linked to tissue and organ fibrosis. These fibrosis‐associated pathways remained elevated on D14 and END (Figure 4a). When considering downregulated genes, during the precachexia phase, we observed enrichment in processes related to oxygen binding and fatty acyl‐coA biosynthesis as early as D8 (Figure 4b), followed by enrichment of carbohydrate metabolism, voltage‐gated potassium channel activity and signalling pathways involved in metabolism, including AMPK and Insulin signalling on D10 (Figure 4b). At the time of cachexia onset (D12), downregulated genes showed enrichment of pathways related to TCA cycle flux, fatty acid β‐oxidation, tRNA biosynthesis, translation elongation and mitochondrial biogenesis (Figure 4b), all of which remained enriched throughout cachexia progression. On D14, downregulated genes became enriched for additional processes related to mitochondrial biogenesis and metabolism, as well as for muscle‐specific genes involved in muscle development, structure and function, which remained enriched at endpoint (END, Figure 4b). To identify potential upstream regulators driving transcriptional changes during the transition from precachexia to cachexia, we performed follow‐up IPA analyses on DEG (Supporting Information File S3). We identified PGC‐1α, let‐7 miRNA, retinoblastoma (RB1), α‐catenin and INSR (insulin receptor) among the top upstream regulators predicted to be *suppressed* in the diaphragm of both precachectic (D10) and cachectic mice (D12) (Figure 5a). These data thus predict early disruptions to energy metabolism and insulin sensitivity/signalling, cellular adhesion and transcriptional pathways governing muscle‐specific gene expression. At these same time points, lipopolysaccharide (LPS), tumour‐necrosis factor (TNF), angiotensin (AGT) and factors known to be released from macrophages and α‐granulates of activated platelets (TGF‐B1 and PDGF BB) were predicted as top upstream regulators *activated* within the diaphragm (Figure 5b), implicating their potential involvement in driving downstream processes that promote diaphragm wasting and fibrotic remodelling.

### 3.5 Hierarchical Clustering Reveals Gene Sets With Similar Temporal Regulation Throughout Cachexia Progression

**FIGURE 6:**
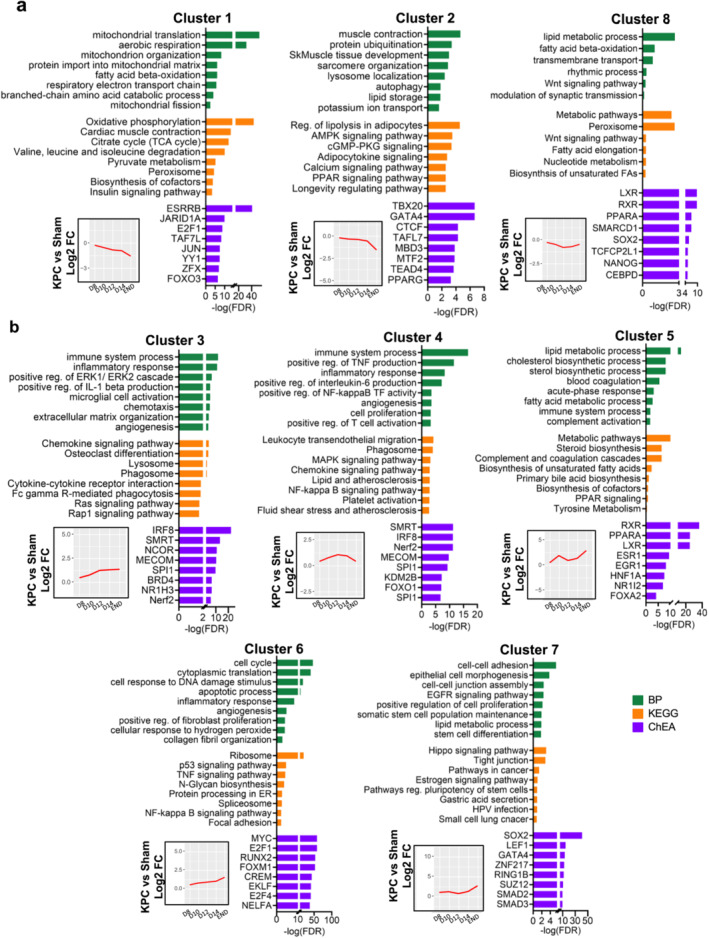
Hierarchical clustering of diaphragm RNAseq data reveals gene clusters with unique biological functions and temporal dynamics throughout PDAC cachexia progression. Hierarchical clustering analysis performed on genes differentially expressed (DEG, padj. <0.01) in the diaphragm of KPC mice (vs. Sham) at one time point or more revealed eight major gene clusters with unique temporal profiles. Bioinformatic enrichment analyses using DAVID and Enrichr were performed on each gene cluster to identify top nonredundant enriched biological processes (DAVID), KEGG pathways (DAVID) and ChEA signature profiles (Enrichr) on clusters showing an overall upregulation (a) or downregulation (b) throughout the cachexia trajectory. Insets depict the average gene expression in diaphragm of KPC mice vs. Sham (red lines) for each cluster on D8, D10, D12, D14 and END.

To identify gene sets and biological processes showing similar temporal regulation throughout the different stages of PDAC cachexia, we performed hierarchical clustering analysis of genes differentially expressed at one time point or more. This analysis revealed eight major gene clusters with unique temporal expression patterns (Figures 6 and S6 and File S4). Enrichment analyses were performed on each cluster using DAVID and Enrichr to identify enriched biological processes and KEGG pathways, potential upstream transcriptional regulators and cell type signatures (Figure 6 and Supporting Information Files S5–S7).

Clusters 1 and 2 demonstrated gene expression patterns that gradually decrease throughout the stages of cachexia (Figure 6a). Among the 2450 genes in Cluster 1, there was an enrichment in processes related to mitochondrial structure and function and oxidative metabolism, which aligns with previous results in other preclinical models of cancer cachexia [29, 30, 31]. Transcription factor enrichment analyses of Cluster 1 genes using ChEA revealed enrichment of target genes of ESRRB and JARID1A—a histone demethylase that supports transcriptional repression. Cluster 2 (995 genes) showed a slower rate of decline than Cluster 1 and was enriched for processes related to the contractile apparatus and muscle development, including target genes of CTCF, an epigenetic modifier of chromatin and TEAD4, which are both known to regulate muscle‐specific genes. Cluster 2 also showed enrichment of genes related to protein ubiquitination, the autophagy‐lysosome system, circadian rhythm and signalling pathways involved in energy homeostasis, including AMPK, cGMP‐PKG and PPAR signalling. Cluster 8 (347 genes, Figure 6a) similarly showed an overall pattern of gene repression throughout cachexia progression, with peak repression observed at the time of cachexia onset. Cluster 8 was most strongly enriched for target genes of nuclear receptors RXR, LXR and PPARA involved in lipid metabolism, including beta oxidation and lipid biosynthetic processes. Cell type signature analyses of Clusters 1, 2 and 8 support a skeletal muscle cell origin.

Clusters 3–7 encompassed genes showing an overall pattern of gene upregulation (Figure 6b). Genes in Cluster 3 (451 genes) and Cluster 4 (521 genes) showed cell type signatures most strongly associated with monocytes/macrophages and other leukocytes. These clusters showed strong enrichment of genes involved in inflammatory processes, including leukocyte recruitment (i.e., chemokines, complement components, adhesion molecules), cytokine production, ECM remodelling and activation of the NF‐κB and MAPK signalling pathways. Genes annotating to Cluster 5 (240 genes) presented a biphasic expression pattern, with average expression peaking higher than any other cluster during the precachexia phase. Cluster 5 showed strong enrichment for processes related to steroid, cholesterol and membrane lipid biosynthesis—processes known to support cellular proliferation and macrophage activation. Cluster 5 was also enriched for processes involved in hemostatic and inflammatory responses to tissue injury, including blood coagulation and complement activation.

Cluster 6 genes (2184 genes) showed a slow but steady increase in their expression, peaking at the advanced cachexia stage when diaphragms showed significant ECM expansion and collagen deposition. Cluster 6 genes were enriched for processes related to wound healing and fibrosis that are known to be regulated via TGF‐β1, including myofibroblast activation, ECM degradation and expansion and the transition of cells to a mesenchymal phenotype (e.g., *Tgfb1, Vim, Snai1, Fn1, Acta2*). Cluster 6 also included marker genes indicative of endothelial inflammation/dysfunction and increased vascular permeability (e.g., *Selp, Icam1, Vcam1*) which were elevated prior to cachexia development. These processes, which can be activated by systemic and local inflammatory mediators, facilitate leukocyte extravasation and contribute to the efflux of fluid and plasma proteins out of tissue capillaries into the interstitial space. Aligning with these findings, endothelial dysfunction and increased vascular permeability have been documented within skeletal muscles of precachectic mice with PDAC [32], supporting the involvement of these processes in leukocyte trafficking to the diaphragm.

Genes annotating to Cluster 7 (1066 genes) showed relatively stable expression until tumour endpoint, when average expression dramatically increased. Cluster 7 genes were enriched for processes/pathways related to wound healing, cell adhesion and cell proliferation and differentiation, including the Hippo and EGFR signalling pathways—which are also linked to tumorigenesis and to PDAC [33, 34]. Cell type analyses of Cluster 7 supported an epithelial origin. Since some diaphragm muscles of moderately/severely cachectic KPC mice (i.e., D14 and END) showed heterogeneous clusters of mononuclear cells located along the inferior perimysium (synonymous with the peritoneum, Figure 2e) which could be indicative of metastases, we extracted two pancreatic cell marker genes from our RNAseq dataset (*Pdx1 and Slc44a4*). Both transcripts were detected in diaphragm muscles by D10 and showed large increases in their abundance at endpoint (Figure S7a), suggesting that PDAC metastasis to the diaphragm could drive Cluster 7 gene expression changes. In line with this finding, metastases have been reported in diaphragm muscles of genetically engineered mouse models of PDAC [35, 36]. However, activation and mesenchymal transition of mesothelial cells—which form the monolayer separating the diaphragm from the peritoneal cavity (i.e., peritoneal mesothelium/peritoneum)—could also be involved in Cluster 7 changes. Mesothelial cells are epithelial in nature, are well‐established to modulate inflammatory, repair and fibrotic responses to tissue injury, and are suspected to contribute to peritoneal metastasis (reviewed in [37, 38]). In support of this latter possibility, mesothelial cell markers (*Pdpn and Wt1)* were also elevated in diaphragms of KPC mice at one time point or more (Figure S7b). Future studies investigating the transcriptome of individual cell types present in diaphragm muscles of control and tumour‐bearing mice using single nucleus RNA‐Seq are thus warranted to validate the findings herein.

### 3.6 Transcriptomic Data Support the Unfolded Protein Response and Muscle Denervation as Potential Mediators of Diaphragm Wasting and Remodelling

**FIGURE 7:**
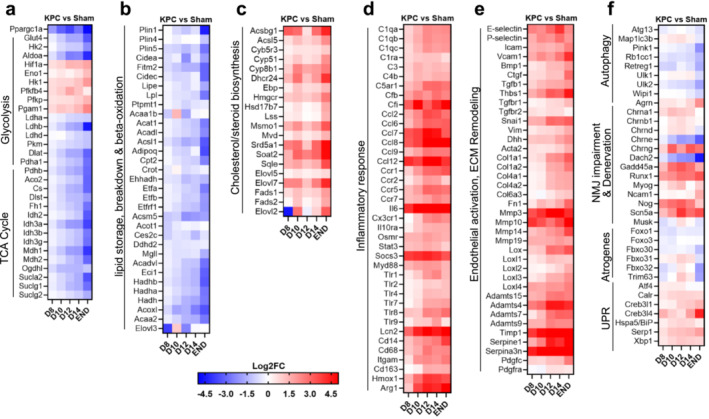
Heatmaps depicting gene expression changes for pathways linked to diaphragm wasting and pathological remodelling prior to and throughout KPC‐induced cachexia. Manually curated genes of interest from the RNAseq dataset showing differential expression of genes involved in glucose uptake, glycolysis and oxidative metabolism (a), lipid storage and breakdown (b), cholesterol/steroid biosynthesis (c), inflammation (d), endothelial dysfunction and ECM remodelling (e) and muscle atrophy‐related pathways (f), including autophagy, neuromuscular junction (NMJ) remodelling associated with denervation, ubiquitin‐dependent protein degradation, endoplasmic reticulum stress and the unfolded protein response (UPR). All data represent Log2FC vs. Sham.

Heatmaps showing expression changes of genes of interest are shown in Figure 7, including genes involved in glucose and oxidative metabolism (Figure 7a), lipid storage and breakdown (Figure 7b), cholesterol/steroid and lipid biosynthesis (Figure 7c), inflammation (Figure 7d), endothelial activation/injury and ECM remodelling (Figure 7e). To obtain further insight into processes established to be involved in muscle wasting, we manually curated genes from our RNAseq dataset (Figure 7f). The diaphragm showed significant repression of FoxO factors, and repression of several genes involved in autophagy, though other autophagy genes were increased. Ubiquitin E3 ligases, *Fbxo32*/atrogin‐1/Mafbx and *Trim63*/MuRF1, were increased in the diaphragm at the time of cachexia onset (D12), supporting their involvement in muscle fibre atrophy, but declined thereafter, showing significant transcriptional repression at endpoint. In contrast, genes linked to the unfolded protein response (UPR), a highly ubiquitous metabolic stress signalling cascade, were activated in diaphragm muscles prior to and throughout cachexia onset, including those linked to the PERK and IRE1α arms of the UPR (Figure 7f). Although targeted ablation of the PERK arm of the UPR exacerbates cancer cachexia in mice [39], inhibition of XBP1, a downstream target of TLR‐MYD88 signalling and the IRE1α arm of the UPR ameliorated cancer‐induced muscle wasting [40]. In support of the latter axis as a potential driver of KPC‐induced wasting in the diaphragm, IPA analyses predicted TLR, MYD88 and XBP1 as upstream regulators activated in diaphragm of precachectic and cachectic KPC mice (Supporting Information File S3). We also observed transcriptional changes associated with neuromuscular junction impairments and denervation in diaphragms of precachectic and cachectic mice, including a prominent upregulation of BMP inhibitor, Noggin, as early as D8. Recent findings have established a key role for Noggin and perturbation of BMP signalling as a mechanism contributing to denervation and cancer‐induced muscle loss [41], thereby supporting this axis as a potential contributor to diaphragm wasting in response to PDAC.

## 4 Conclusion

**FIGURE 8:**
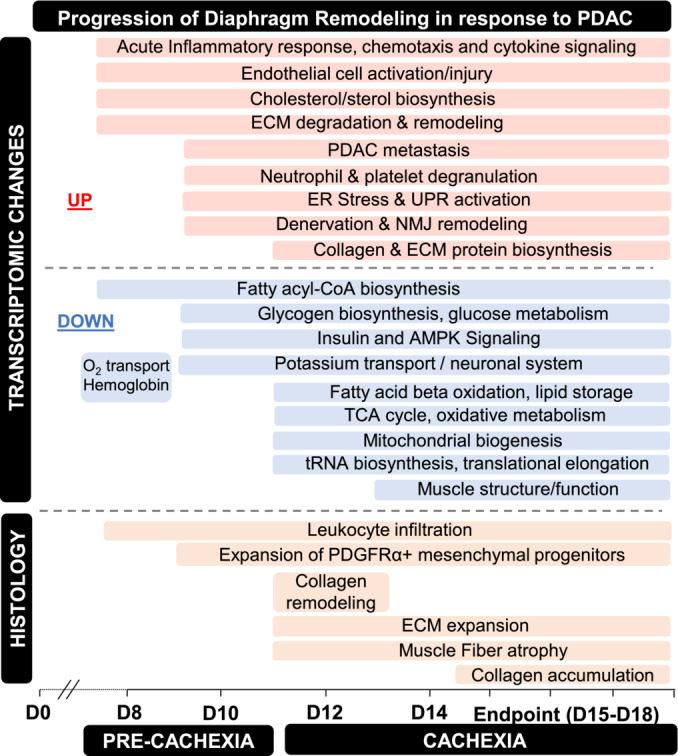
Schematic summary depicting the sequence of events linked to muscle wasting and pathological remodelling of the diaphragm in response to pancreatic cancer. The events depicted are based upon transcriptomic or histological analyses of the mouse diaphragm at various time points throughout cachexia progression in an orthotopic model of PDAC that utilises murine KPC cells. D0–18 = 0–18 days after surgery, ECM = extracellular matrix, ER = endoplasmic reticulum, NMJ = neuromuscular junction.

In summary, our findings reveal leukocyte infiltration and inflammatory processes within the mouse diaphragm as key events during the precachexia stage of PDAC that precedes the expansion of PDGFR‐α^+^ mesenchymal progenitors, muscle wasting and fibrosis development (schematic presented in Figure 8). Importantly, local inflammation can also cause major shifts in parenchymal tissue metabolism that can disrupt tissue homeostasis. Such changes include local depletion of nutrients and oxygen due to their increased consumption by infiltrating leukocytes and proliferating cells, and increased production of reactive oxygen species that can further promote tissue damage (reviewed in [42]). Thus, inflammation could also underlie the transcriptional reprogramming of metabolic networks observed in the diaphragm throughout cachexia development and progression. Based on the collective findings herein, we therefore posit that metabolic stress induced by local inflammation may represent a key driver of respiratory muscle wasting and fibrotic remodelling in response to PDAC that warrants further study.

## Conflicts of Interest

The authors declare no conflicts of interest.

## Code Statement

No custom algorithm was used for this manuscript. Scripts used to perform RNAseq follow‐up analyses are available upon request.

## Supporting information


**Figure S1** Progressive cardiac wasting and splenomegaly throughout the development and progression of KPC‐induced cachexia. (a) Schematic representation of the experimental design, showing postsurgery time points (Day 8, Day 10, Day 12, Day 14 and END) at which diaphragm muscles, skeletal muscle, heart, fat, spleen and liver were harvested following surgical isolation and injection of the mouse pancreas with murine pancreatic cancer cells (KPC) or saline (Sham). (b–d) Exponential pancreatic KPC tumours growth associates with cardiac wasting (c) and splenomegaly (d). Livermass (e) was not statistically changed. All *p*‐values <0.1 are included, symbols represent individual data and bars represent mean ± SE.
**Figure S2** Sham abdominal surgeries induce significant fat wasting with in the first week following surgery. Gonadal fat mass was harvested from Sham mice 8–10 days (D8–10) and 14–15 days (D14–15) following sham surgeries and from age‐matched controls that did not undergo surgery (No‐Sx). For all mice, fat mass was normalised to body mass at the start of the experiment (D0). Data points for Sham mice (D14‐D15) are duplicated from the main figure. Data represent mean ± SE.
**Figure S3** Temporal changes in circulating factors in response to KPC tumour burden. Temporal changes in the concentrations of KC (a), IL‐5 (b), IL‐6 (c), IP‐10 (d) and G‐CSF (e) in response to KPC tumour burden as measured through a 25‐plex Luminex panel. Only factors showing significant differences at one time point or more are shown. All *p*‐values <0.1 are included, symbols represent individual data and bars represent mean ± SE.
**Figure S4** Predominant atrophy of Type II A and Type IIX/B myofibers and shift toward Type I and II A myofibers throughout the cachexia trajectory in diaphragm muscles of KPC mice. (a‐c) Quantification of fibre‐type specific diaphragm minimum Feret diameter (MFD) reveals KPC‐induced atrophy of Type II A (a) and Type IIX/B myofibers (b), but not Type I (c) myofibers. (d‐f) KPC–induced type IIA and IIX/B fibre atrophy associates with alterations in fibre‐type composition. MyHC = myosin heavy chain. All *p*‐values <0.1 are included, symbols represent individual data and bars represent mean ± SE.
**Figure S5** Principal component analysis reveals good separation between time points. PC1 = principal component 1, PC2 = principal component 2, SHAM = diaphragms from mice that underwent sham surgery (i.e., nontumour‐bearing mice), D8–14 = diaphragms from KPC tumour‐bearing mice harvested 8–14 days post KPC cell injection, END = diaphragms from KPC tumour‐bearing mice harvested at humane endpoint.
**Figure S6** Hierarchical clustering identifies gene clusters with unique temporal dynamics throughout cachexia progression. (a) Hierarchical clustering analysis performed on genes differentially expressed in the diaphragm of KPC mice (vs. Sham) at one time point or more revealed eight major gene clusters with unique temporal profiles. Individual (blacklines) and averaged (redlines) gene expression changes are shown for genes within each cluster. (b) Plots demonstrating the number of DEGs (padj < 0.01) with in a given cluster at each time point.
**Figure S7** Marker genes of pancreatic progenitors and mesothelial cells are detected in diaphragm of precachectic KPC mice. (a) Gene markers for pancreatic progenitors extracted from Diaphragm RNAseq data, demonstrating detectable levels of expression between D8 and D14 that dramatically increase at humane endpoint when mice are severely cachectic. (b) Gene markers for mesothelial cells, which are also of epithelial origin extracted from Diaphragm RNAseq data, demonstrating detectable levels in Sham mice and increased levels in KPC mice. All statistics correspond to adjusted *p*‐values obtained from the RNAseq analyses, comparing each individual KPC time point to Sham. Data represent mean SE.

**Data S1** Supporting Information

**Data S2** Supporting Information

**Data S3** Supporting Information

**Data S4** Supporting Information

**Data S5** Supporting Information

**Data S6** Supporting Information

**Data S7** Supporting Information

**Data S8** Supporting Information

**Data S9** Supporting Information

## Data Availability

The authors declare that all data generated or analysed in this study are either (i) deposited to Gene Expression Omnibus (www. ncbi.nlm.nih.gov/geo/) or (ii) included in this published article and its supplementary information files. The RNAseq data are accessible with the identifiers GSE271521. The data used to generate figures are provided in Supporting Information File S8.

## References

[1] K. Fearon , J. Arends , and V. Baracos , “Understanding the Mechanisms and Treatment Options in Cancer Cachexia,” Nature Reviews Clinical Oncology 10 (2013): 90–99. 10.1038/nrclinonc.2012.209 23207794

[2] J. Bachmann , M. Heiligensetzer , H. Krakowski‐Roosen , M. W. Buchler , H. Friess , and M. E. Martignoni , “Cachexia Worsens Prognosis in Patients With Resectable Pancreatic Cancer,” Journal of Gastrointestinal Surgery 12 (2008): 1193–1201. 18347879 10.1007/s11605-008-0505-z

[3] J. M. Argiles , B. Stemmler , F. J. Lopez‐Soriano , and S. Busquets , “Inter‐Tissue Communication in Cancer Cachexia,” Nature Reviews. Endocrinology 15 (2018): 9–20. 10.1038/s41574-018-0123-0 30464312

[4] V. E. Baracos , L. Martin , M. Korc , D. C. Guttridge , and K. C. H. Fearon , “Cancer‐Associated Cachexia,” Nature Reviews Disease Primers 4 (2018): 17105. 10.1038/nrdp.2017.105 29345251

[5] L. Nemer , S. G. Krishna , Z. K. Shah , et al., “Predictors of Pancreatic Cancer‐Associated Weight Loss and Nutritional Interventions,” Pancreas 46 (2017): 1152–1157. 28902785 10.1097/MPA.0000000000000898 PMC5679236

[6] S. M. Judge , R. L. Nosacka , D. Delitto , et al., “Skeletal Muscle Fibrosis in Pancreatic Cancer Patients With Respect to Survival,” JNCI Cancer Spectrum. 2 (2019): pky043. 10.1093/jncics/pky043 PMC6322478 30637373

[7] M. R. Deyhle , C. S. Callaway , D. Neyroud , A. C. D'Lugos , S. M. Judge , and A. R. Judge , “Depleting Ly6G Positive Myeloid Cells Reduces Pancreatic Cancer‐Induced Skeletal Muscle Atrophy,” Cells 11 (2022): 11. 10.3390/cells11121893 PMC9221479 35741022

[8] D. Wang , X. Li , D. Jiao , et al., “LCN2 Secreted by Tissue‐Infiltrating Neutrophils Induces the Ferroptosis and Wasting of Adipose and Muscle Tissues in Lung Cancer Cachexia,” Journal of Hematology & Oncology 16 (2023): 30. 36973755 10.1186/s13045-023-01429-1 PMC10044814

[9] A. Anoveros‐Barrera , A. S. Bhullar , C. Stretch , et al., “Immunohistochemical Phenotyping of T Cells, Granulocytes, and Phagocytes in the Muscle of Cancer Patients: Association With Radiologically Defined Muscle Mass and Gene Expression,” Skeletal Muscle 9 (2019): 24. 31521204 10.1186/s13395-019-0209-y PMC6744687

[10] S. K. Shukla , S. D. Markov , K. S. Attri , et al., “Macrophages Potentiate STAT3 Signaling in Skeletal Muscles and Regulate Pancreatic Cancer Cachexia,” Cancer Letters 484 (2020): 29–39. 32344015 10.1016/j.canlet.2020.04.017 PMC7286478

[11] T. A. Washington , E. R. Schrems , W. S. Haynie , et al., “Development of Skeletal Muscle Fibrosis in a Rodent Model of Cancer Cachexia,” Cell Biochemistry and Function 41 (2023): 478–489. 37150891 10.1002/cbf.3797 PMC10330674

[12] R. L. Nosacka , A. E. Delitto , D. Delitto , et al., “Distinct Cachexia Profiles in Response to Human Pancreatic Tumours in Mouse Limb and Respiratory Muscle,” Journal of Cachexia, Sarcopenia and Muscle 11 (2020): 820–837. 32039571 10.1002/jcsm.12550 PMC7296265

[13] N. Sivaram , P. A. McLaughlin , H. V. Han , et al., “Tumor‐Intrinsic PIK3CA Represses Tumor Immunogenecity in a Model of Pancreatic Cancer,” Journal of Clinical Investigation 129 (2019): 3264–3276. 31112530 10.1172/JCI123540 PMC6668699

[14] D. Delitto , S. M. Judge , A. E. Delitto , et al., “Human Pancreatic Cancer Xenografts Recapitulate key Aspects of Cancer Cachexia,” Oncotarget 8 (2017): 1177–1189. 27901481 10.18632/oncotarget.13593 PMC5352045

[15] N. P. Whitehead , K. L. Bible , M. J. Kim , G. L. Odom , M. E. Adams , and S. C. Froehner , “Validation of Ultrasonography for non‐Invasive Assessment of Diaphragm Function in Muscular Dystrophy,” Journal of Physiology 594 (2016): 7215–7227. 27570057 10.1113/JP272707 PMC5157096

[16] O. Laitano , G. P. Robinson , C. K. Garcia , et al., “Skeletal Muscle Interleukin‐6 Contributes to the Innate Immune Response in Septic Mice,” Shock 55 (2021): 676–685. 32826815 10.1097/SHK.0000000000001641 PMC8607997

[17] M. K. Abramowitz , W. Paredes , K. Zhang , et al., “Skeletal Muscle Fibrosis Is Associated With Decreased Muscle Inflammation and Weakness in Patients With Chronic Kidney Disease,” American Journal of Physiology. Renal Physiology 315 (2018): F1658–F1669. 30280599 10.1152/ajprenal.00314.2018 PMC6336993

[18] D. Kopinke , E. C. Roberson , and J. F. Reiter , “Ciliary Hedgehog Signaling Restricts Injury‐Induced Adipogenesis,” Cell 170 (2017): 340–351.e12. 28709001 10.1016/j.cell.2017.06.035 PMC5617351

[19] D. Neyroud , O. Laitano , A. Dasgupta , et al., “Blocking Muscle Wasting via Deletion of the Muscle‐Specific E3 Ligase MuRF1 Impedes Pancreatic Tumor Growth,” Communications Biology 6 (2023): 519. 37179425 10.1038/s42003-023-04902-2 PMC10183033

[20] E. Azoulay , G. Thiery , S. Chevret , et al., “The Prognosis of Acute Respiratory Failure in Critically ill Cancer Patients,” Medicine (Baltimore) 83 (2004): 360–370. 15525848 10.1097/01.md.0000145370.63676.fb

[21] B. Raynard , F. Pigneur , M. Di Palma , E. Deluche , and F. Goldwasser , “The Prevalence of CT‐Defined Low Skeletal Muscle Mass in Patients With Metastatic Cancer: A Cross‐Sectional Multicenter French Study (The SCAN Study),” Supportive Care in Cancer: Official Journal of the Multinational Association of Supportive Care in Cancer 30, no. 4 (2022): 3119–3129, 10.1007/s00520-021-06603-0. 34862578 PMC8857123

[22] L. J. Delfinis , C. A. Bellissimo , S. Gandhi , et al., “Muscle Weakness Precedes Atrophy During Cancer Cachexia and Is Linked to Muscle‐Specific Mitochondrial Stress,” JCI Insight 7 (2022): 7. 10.1172/jci.insight.155147 PMC9869968 36346680

[23] S. M. Judge , M. R. Deyhle , D. Neyroud , et al., “MEF2c‐Dependent Downregulation of Myocilin Mediates Cancer‐Induced Muscle Wasting and Associates With Cachexia in Patients With Cancer,” Cancer Research 80 (2020): 1861–1874. 32132110 10.1158/0008-5472.CAN-19-1558 PMC7250164

[24] J. Hwang , Y. Huang , T. J. Burwell , et al., “In Situ Imaging of Tissue Remodeling With Collagen Hybridizing Peptides,” ACS Nano 11 (2017): 9825–9835. 28877431 10.1021/acsnano.7b03150 PMC5656977

[25] R. L. Lieber and S. R. Ward , “Cellular Mechanisms of Tissue Fibrosis. 4. Structural and Functional Consequences of Skeletal Muscle Fibrosis,” American Journal of Physiology Cell Physiology 305 (2013): C241–C252. 23761627 10.1152/ajpcell.00173.2013 PMC3742845

[26] M. Zeisberg and R. Kalluri , “Cellular Mechanisms of Tissue Fibrosis. 1. Common and Organ‐Specific Mechanisms Associated With Tissue Fibrosis,” American Journal of Physiology Cell Physiology 304 (2013): C216–C225. 23255577 10.1152/ajpcell.00328.2012 PMC3566435

[27] W. A. He , E. Berardi , V. M. Cardillo , et al., “NF‐kappaB‐Mediated Pax7 Dysregulation in the Muscle Microenvironment Promotes Cancer Cachexia,” Journal of Clinical Investigation 123 (2013): 4821–4835. 24084740 10.1172/JCI68523 PMC3809785

[28] A. Uezumi , T. Ito , D. Morikawa , et al., “Fibrosis and Adipogenesis Originate From a Common Mesenchymal Progenitor in Skeletal Muscle,” Journal of Cell Science 124 (2011): 3654–3664. 22045730 10.1242/jcs.086629

[29] F. A. Graca , A. Stephan , Y. D. Wang , et al., “Progressive Development of Melanoma‐Induced Cachexia Differentially Impacts Organ Systems in Mice,” Cell Reports 42 (2023): 111934. 36640353 10.1016/j.celrep.2022.111934 PMC9983329

[30] T. A. Blackwell , I. Cervenka , B. Khatri , et al., “Transcriptomic Analysis of the Development of Skeletal Muscle Atrophy in Cancer‐Cachexia in Tumor‐Bearing Mice,” Physiological Genomics 50 (2018): 1071–1082. 30289747 10.1152/physiolgenomics.00061.2018 PMC6337023

[31] J. L. Brown , M. E. Rosa‐Caldwell , D. E. Lee , et al., “Mitochondrial Degeneration Precedes the Development of Muscle Atrophy in Progression of Cancer Cachexia in Tumour‐Bearing Mice,” Journal of Cachexia, Sarcopenia and Muscle 8 (2017): 926–938. 28845591 10.1002/jcsm.12232 PMC5700433

[32] Y.‐M. Kim , M. A. Sanborn , X. Wang , et al., “Impaired Barrier Integrity of the Skeletal Muscle Vascular Endothelium Drives Progression of Cancer Cachexia,” bioRxiv. (2022) 2022.12.12.520118.

[33] D. Ansari , H. Ohlsson , C. Althini , et al., “The Hippo Signaling Pathway in Pancreatic Cancer,” Anticancer Research 39 (2019): 3317–3321. 31262852 10.21873/anticanres.13474

[34] M. Williams , G. Lomberk , and R. Urrutia , EGFR (ErbB) Signaling Pathways in Pancreatic Cancer Pathogenesis, eds. J. P. Neoptolemos , R. Urrutia , J. L. Abbruzzese , and M. W. Büchler (New York, NY: Springer, 2018): 383–408.

[35] S. R. Hingorani , L. Wang , A. S. Multani , et al., “Trp53R172H and KrasG12D Cooperate to Promote Chromosomal Instability and Widely Metastatic Pancreatic Ductal Adenocarcinoma in Mice,” Cancer Cell 7 (2005): 469–483. 15894267 10.1016/j.ccr.2005.04.023

[36] J. W. Lee , C. A. Komar , F. Bengsch , K. Graham , and G. L. Beatty , “Genetically Engineered Mouse Models of Pancreatic Cancer: The KPC Model (*LSL‐Kras* ^ *G12D*/+^;*LSL‐Trp53* ^R172H/+^;*Pdx‐1‐Cre*), Its Variants, and Their Application in Immuno‐Oncology Drug Discovery,” Current Protocols in Pharmacology 73 (2016): 14.39.1–14.39.20. 10.1002/cpph.2 PMC4915217 27248578

[37] S. E. Mutsaers , K. Birnie , S. Lansley , S. E. Herrick , C. B. Lim , and C. M. Prele , “Mesothelial Cells in Tissue Repair and Fibrosis,” Frontiers in Pharmacology 6 (2015): 113. 26106328 10.3389/fphar.2015.00113 PMC4460327

[38] A. Rynne‐Vidal , J. A. Jimenez‐Heffernan , C. Fernandez‐Chacon , M. Lopez‐Cabrera , and P. Sandoval , “The Mesothelial Origin of Carcinoma Associated‐Fibroblasts in Peritoneal Metastasis,” Cancers 7 (2015): 1994–2011. 26426054 10.3390/cancers7040872 PMC4695872

[39] Y. S. Gallot , K. R. Bohnert , A. R. Straughn , G. Xiong , S. M. Hindi , and A. Kumar , “PERK Regulates Skeletal Muscle Mass and Contractile Function in Adult Mice,” FASEB Journal 33 (2019): 1946–1962. 30204503 10.1096/fj.201800683RR PMC6338633

[40] K. R. Bohnert , P. Goli , A. Roy , et al., “The Toll‐Like Receptor/MyD88/XBP1 Signaling Axis Mediates Skeletal Muscle Wasting During Cancer Cachexia,” Molecular and Cellular Biology 39 (2019): 67–78. 10.1128/MCB.00184-19 PMC6639248 31138662

[41] R. Sartori , A. Hagg , S. Zampieri , et al., “Perturbed BMP Signaling and Denervation Promote Muscle Wasting in Cancer Cachexia,” Science Translational Medicine 13 (2021): 13. 10.1126/scitranslmed.aay9592 34349036

[42] D. J. Kominsky , E. L. Campbell , and S. P. Colgan , “Metabolic Shifts in Immunity and Inflammation,” Journal of Immunology 184 (2010): 4062–4068. 10.4049/jimmunol.0903002 PMC4077461 20368286

