# Supplementary material for: Local Inflammation Precedes Diaphragm Wasting and Fibrotic Remodelling in a Mouse Model of Pancreatic Cancer
Source: J Cachexia Sarcopenia Muscle. 2025 Jan 15;16(1):e13668. doi: 10.1002/jcsm.13668 (PMC11733308; doi:10.1002/jcsm.13668)
Supplement: Supplementary file 1 — Figure S1 Progressive cardiac wasting and splenomegaly throughout the development and progression of KPC‐induced cachexia. (a) Schematic representation of the experimental design, showing postsurgery time points (Day 8, Day 10, Day 12, Day 14 and END) at which diaphragm muscles, skeletal muscle, heart, fat, spleen and liver were harvested following surgical isolation and injection of the mouse pancreas with murine pancreatic cancer cells (KPC) or saline (Sham). (b–d) Exponential pancreatic KPC tumours growth associates with cardiac wasting (c) and splenomegaly (d). Livermass (e) was not statistically changed. All p‐values <0.1 are included, symbols represent individual data and bars represent mean ± SE. Figure S2 Sham abdominal surgeries induce significant fat wasting with in the first week following surgery. Gonadal fat mass was harvested from Sham mice 8–10 days (D8–10) and 14–15 days (D14–15) following sham surgeries and from age‐matched controls that did not undergo surgery (No‐Sx). For all mice, fat mass was normalised to body mass at the start of the experiment (D0). Data points for Sham mice (D14‐D15) are duplicated from the main figure. Data represent mean ± SE. Figure S3 Temporal changes in circulating factors in response to KPC tumour burden. Temporal changes in the concentrations of KC (a), IL‐5 (b), IL‐6 (c), IP‐10 (d) and G‐CSF (e) in response to KPC tumour burden as measured through a 25‐plex Luminex panel. Only factors showing significant differences at one time point or more are shown. All p‐values <0.1 are included, symbols represent individual data and bars represent mean ± SE. Figure S4 Predominant atrophy of Type II A and Type IIX/B myofibers and shift toward Type I and II A myofibers throughout the cachexia trajectory in diaphragm muscles of KPC mice. (a‐c) Quantification of fibre‐type specific diaphragm minimum Feret diameter (MFD) reveals KPC‐induced atrophy of Type II A (a) and Type IIX/B myofibers (b), but not Type I (c) myofibers. (d‐f) KPC [file JCSM-16-e13668-s005.pdf]

A

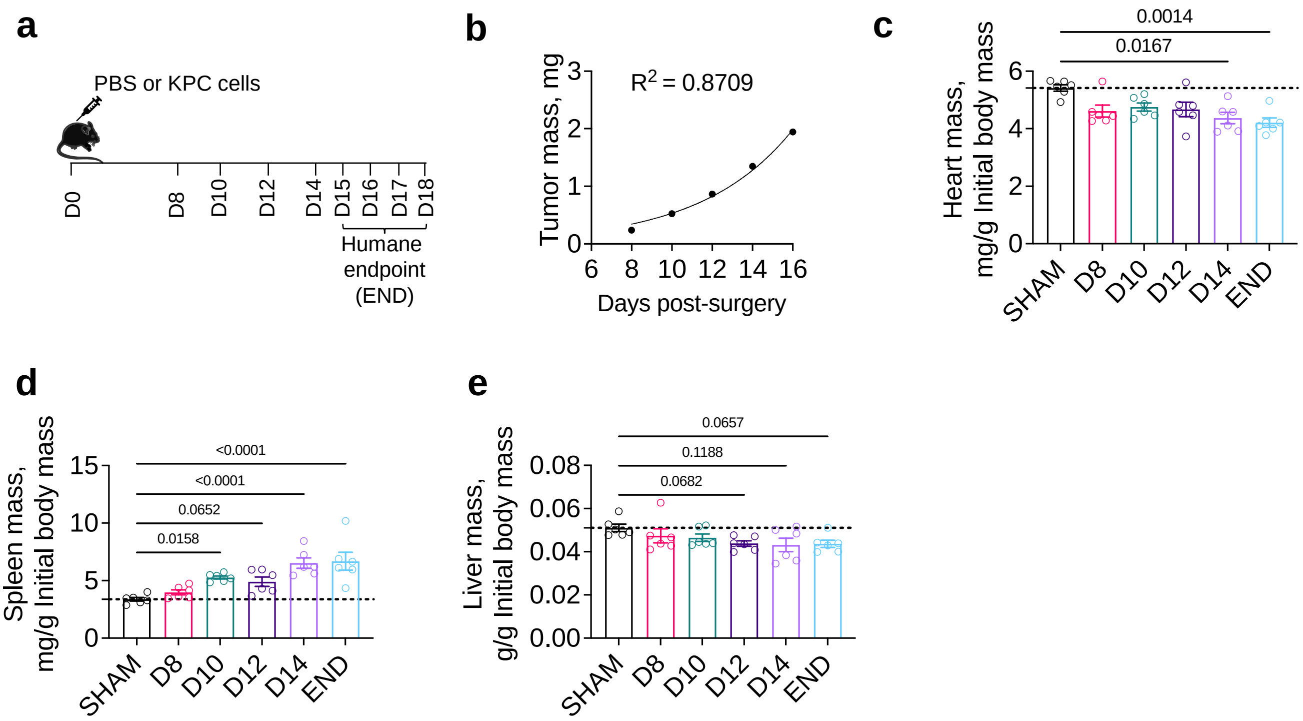

Supplementary Fig. S1 Progressive cardiac wasting and splenomegaly throughout the development and progression of KPC-induced cachexia. (**a**) Schematic representation of the experimental design, showing post-surgery time points (Day 8, Day 10, Day 12, Day 14 and END) at which diaphragm muscles, skeletal muscle, heart, fat, spleen and liver were harvested following surgical isolation and injection of the mouse pancreas with murine pancreatic cancer cells (KPC) or saline (Sham). (**b-d**) Exponential pancreatic KPC tumors growth associates with cardiac wasting (**c**) and splenomegaly (**d**). Liver mass (**e**) was not statistically changed. All P-values < 0.1 are included, symbols represent individual data, and bars represent mean ± SE.

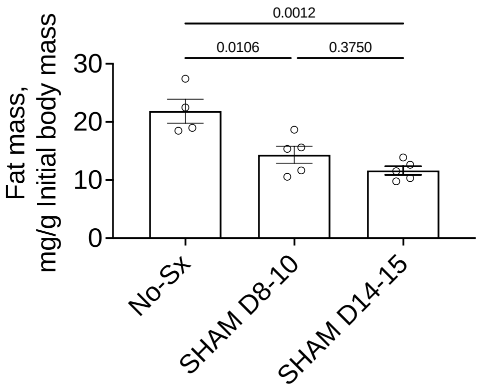

Supplementary Fig. S2 Sham abdominal surgeries induce significant fat wasting within the first week following surgery

Gonadal fat mass was harvested from Sham mice 8-10 days (D8-10) and 14-15 days (D14-15) following sham surgeries, and from age-matched controls that did not undergo surgery (No-Sx). For all mice, fat mass was normalized to body mass at the start of the experiment (D0). Data points for Sham mice (D14-D15) are duplicated from the main figure. Data represent mean ± SE.

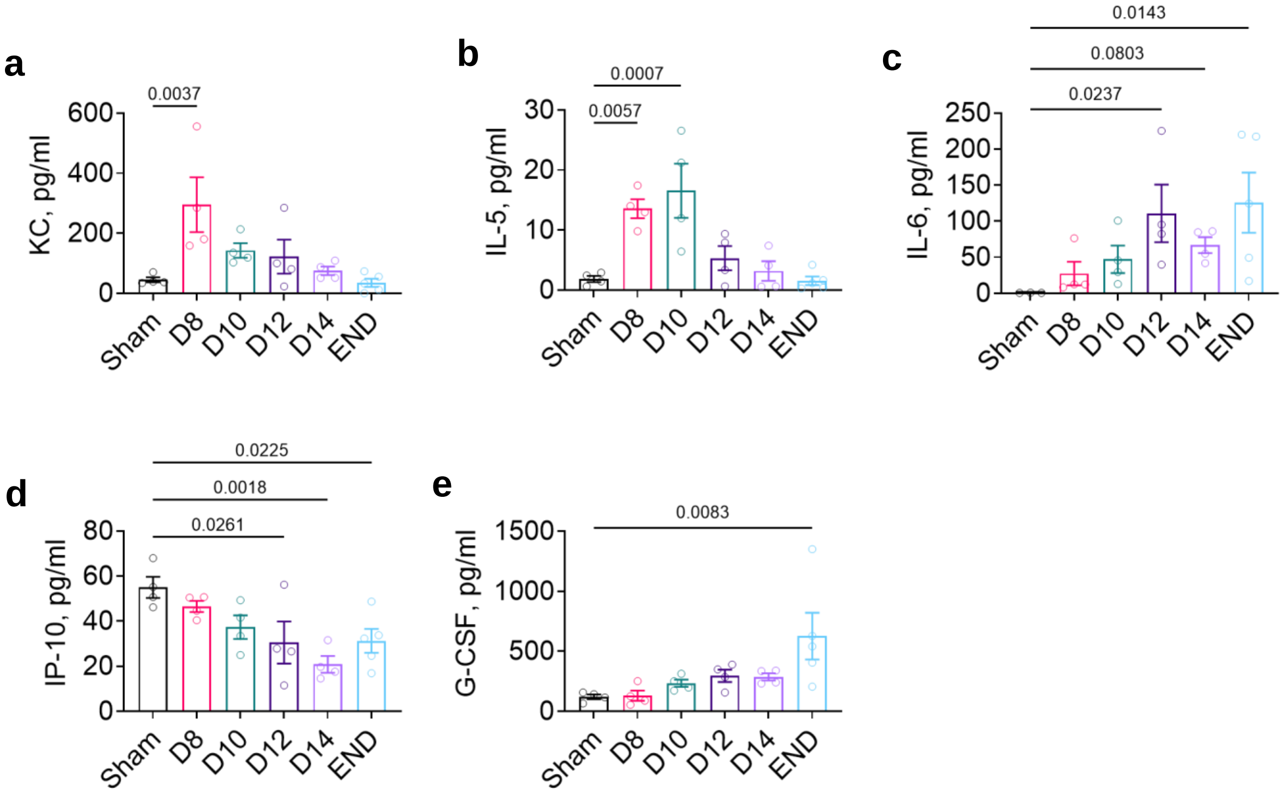

Supplementary Fig. S3 Temporal changes in circulating factors in response to KPC tumor burden

Temporal changes in the concentrations of KC (**a**), IL-5 (**b**), IL-6 (**c**), IP-10 (**d**) and G-CSF (**e**) in response to KPC tumor burden as measured through a 25-plex Luminex panel. Only factors showing significant differences at one time point or more are shown. All P-values < 0.1 are included, symbols represent individual data, and bars represent mean ± SE.

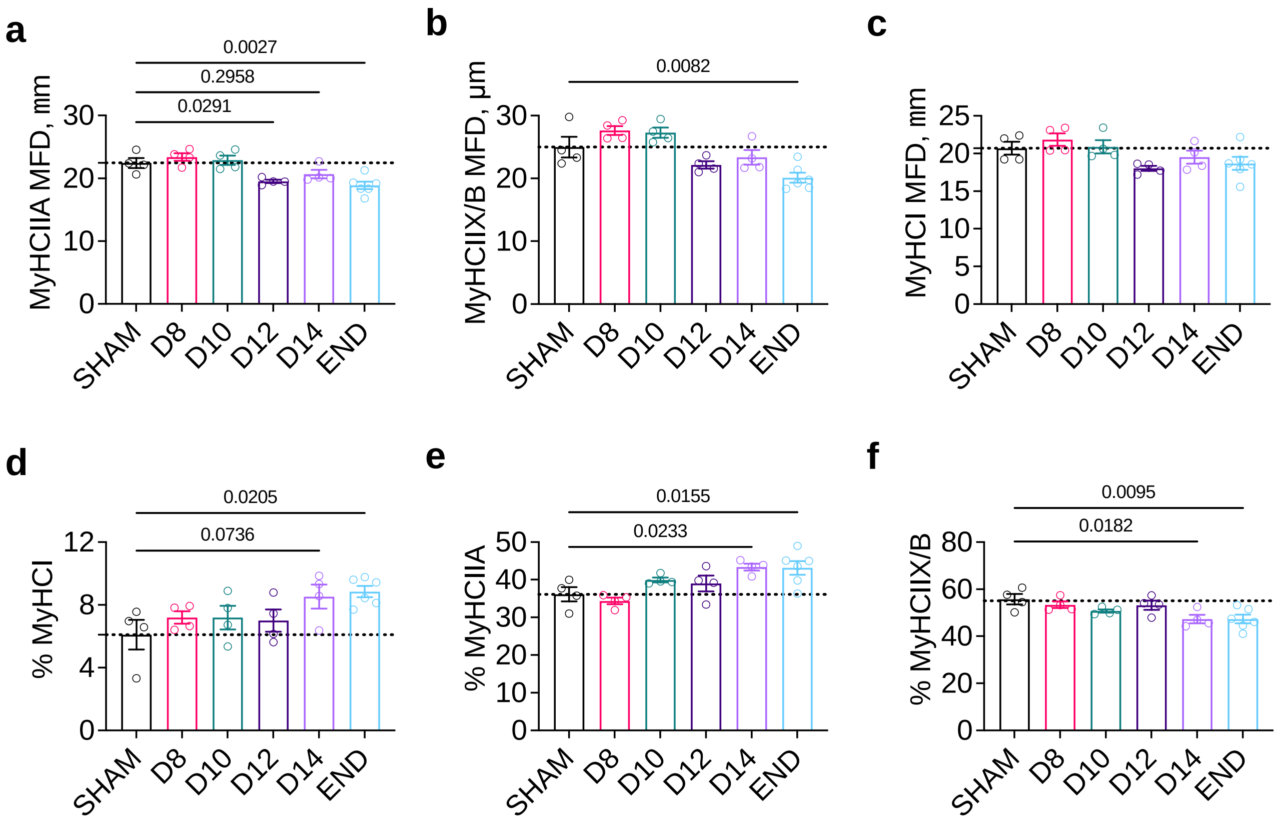

Supplementary Fig. S4 Predominant atrophy of Type IIA and Type IIX/B myofibers and shift toward Type I and IIA myofibers throughout the cachexia trajectory in diaphragm muscles of KPC mice

(**a-c**) Quantification of fiber-type specific diaphragm minimum Feret diameter (MFD) reveals KPC-induced atrophy of Type IIA (**a**) and Type IIX/B myofibers (**b**), but not Type I (**c**) myofibers. (**d-f**) KPC–induced type IIA and IIX/B fiber atrophy associates with alterations in fiber type composition. MyHC = myosin heavy chain. All P-values < 0.1 are included, symbols represent individual data, and bars represent mean ± SE.

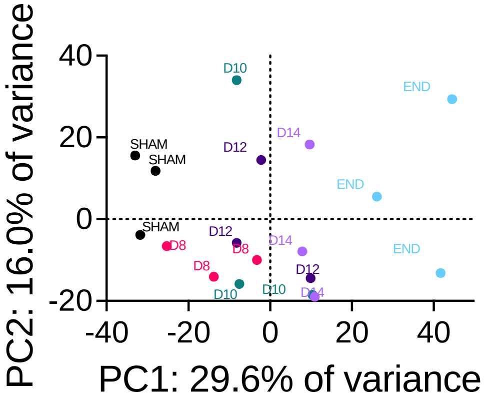

Supplementary Fig. S5 Principal component analysis reveals good separation between time points

PC1 = principal component 1, PC2 = principal component 2, SHAM = diaphragms from mice that underwent sham surgery (i.e. non-tumor-bearing mice), D8-14 = diaphragms from KPC tumor-bearing mice harvested 8-14 days post KPC cell injection, END = diaphragms from KPC tumor-bearing mice harvested at humane endpoint.

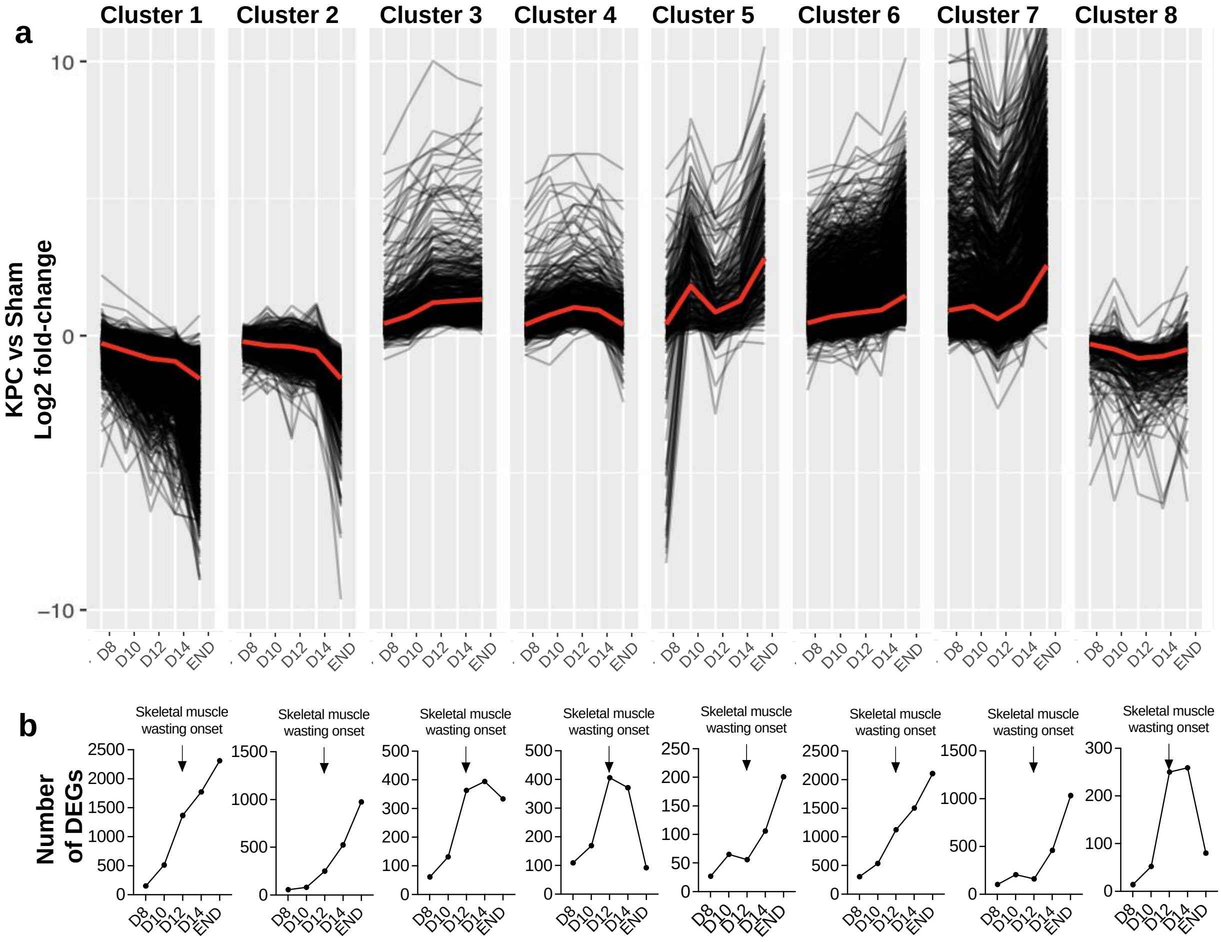

Supplementary Fig. S6 Hierarchical clustering identifies gene clusters with unique temporal dynamics throughout cachexia progression

(**a**) Hierarchical clustering analysis performed on genes differentially expressed in the diaphragm of KPC mice (vs Sham) at one time point or more revealed 8 major gene clusters with unique temporal profiles. Individual (black lines) and averaged (red lines) gene expression changes are shown for genes within each cluster. (**b**) Plots demonstrating the number of DEGs (padj < 0.01) within a given cluster at each time point.

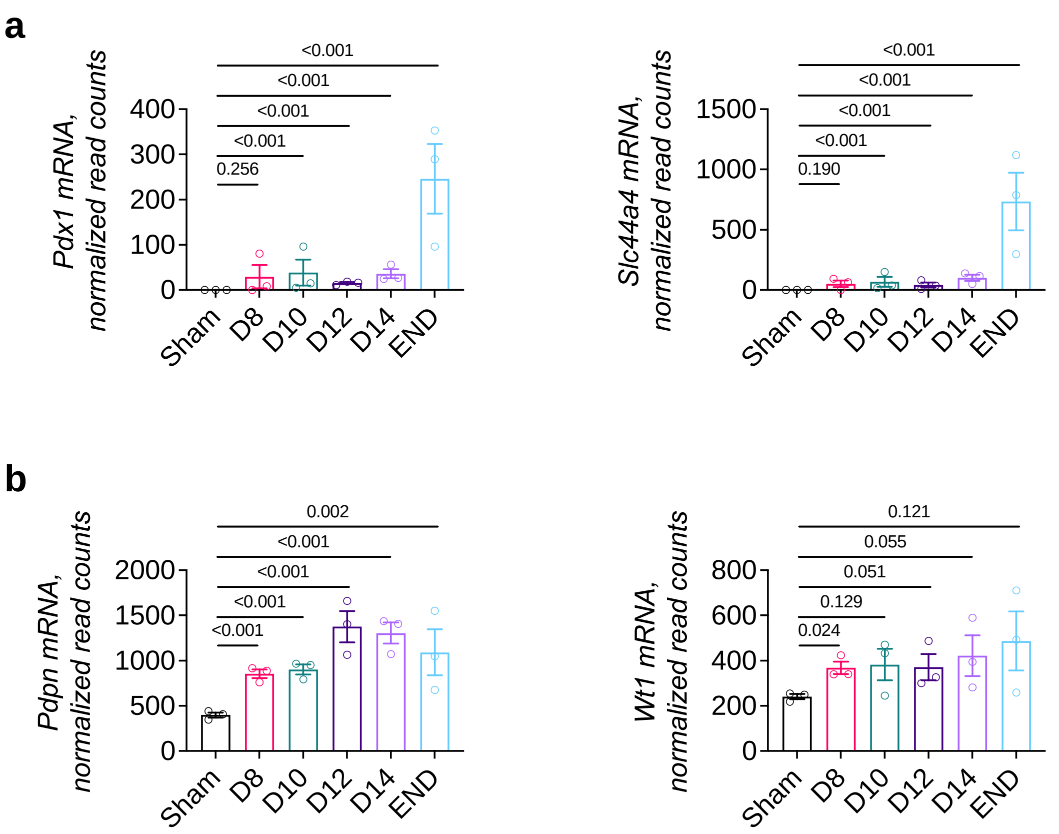

Supplementary Fig. S7 Marker genes of pancreatic progenitors and mesothelial cells are detected in diaphragm of pre-cachectic KPC mice

(**a**) Gene markers for pancreatic progenitors extracted from Diaphragm RNAseq data, demonstrating detectable levels of expression between D8-D14 that dramatically increase at humane endpoint when mice are severely cachectic. (**b**) Gene markers for mesothelial cells, which are also of epithelial origin extracted from Diaphragm RNAseq data, demonstrating detectable levels in Sham mice and increased levels in KPC mice. All statistics correspond to adjusted p-values obtained from the RNAseq analyses, comparing each individual KPC timepoint to Sham. Data represent mean SE.
