# Supplementary material for: Local Inflammation Precedes Diaphragm Wasting and Fibrotic Remodelling in a Mouse Model of Pancreatic Cancer
Source: J Cachexia Sarcopenia Muscle. 2025 Jan 15;16(1):e13668. doi: 10.1002/jcsm.13668 (PMC11733308; doi:10.1002/jcsm.13668)
Supplement: Supplementary file 10 — Data S9 Supporting Information [file JCSM-16-e13668-s002.docx]

**Supplementary References**

1. Senf SM, Howard TM, Ahn B, Ferreira LF, Judge AR. Loss of the inducible Hsp70 delays the inflammatory response to skeletal muscle injury and severely impairs muscle regeneration. PloS one. 2013;8:e62687.

2. Schindelin J, Arganda-Carreras I, Frise E, Kaynig V, Longair M, Pietzsch T, et al. Fiji: an open-source platform for biological-image analysis. Nat Methods. 2012;9:676-82.

3. Nakae Y, Dorchies OM, Stoward PJ, Zimmermann BF, Ritter C, Ruegg UT. Quantitative evaluation of the beneficial effects in the mdx mouse of epigallocatechin gallate, an antioxidant polyphenol from green tea. Histochemistry and cell biology. 2012;137:811-27.

4. Kramer A, Green J, Pollard J, Jr., Tugendreich S. Causal analysis approaches in Ingenuity Pathway Analysis. Bioinformatics. 2014;30:523-30.

5. Sherman BT, Hao M, Qiu J, Jiao X, Baseler MW, Lane HC, et al. DAVID: a web server for functional enrichment analysis and functional annotation of gene lists (2021 update). Nucleic acids research. 2022;

6. Huang da W, Sherman BT, Lempicki RA. Systematic and integrative analysis of large gene lists using DAVID bioinformatics resources. Nature protocols. 2009;4:44-57.

7. Kuleshov MV, Jones MR, Rouillard AD, Fernandez NF, Duan Q, Wang Z, et al. Enrichr: a comprehensive gene set enrichment analysis web server 2016 update. Nucleic acids research. 2016;44:W90-7.

8. Chen EY, Tan CM, Kou Y, Duan Q, Wang Z, Meirelles GV, et al. Enrichr: interactive and collaborative HTML5 gene list enrichment analysis tool. BMC Bioinformatics. 2013;14:128.

9. Xie Z, Bailey A, Kuleshov MV, Clarke DJB, Evangelista JE, Jenkins SL, et al. Gene Set Knowledge Discovery with Enrichr. Curr Protoc. 2021;1:e90.
